# Recent Advances in Ultralow‐Pt‐Loading Electrocatalysts for the Efficient Hydrogen Evolution

**DOI:** 10.1002/advs.202301098

**Published:** 2023-05-10

**Authors:** Fei Guo, Thomas J. Macdonald, Ana Jorge Sobrido, Longxiang Liu, Jianrui Feng, Guanjie He

**Affiliations:** ^1^ Department of Chemical Engineering University College London London WC1E 7JE UK; ^2^ Materials Research Institute School of Engineering and Materials Science Faculty of Science and Engineering Queen Mary University of London Mile End Road London E1 4NS UK

**Keywords:** electrocatalysts, hydrogen evolution reaction, ultralow‐Pt‐loading

## Abstract

Hydrogen production from water electrolysis provides a green and sustainable route. Platinum (Pt)‐based materials have been regarded as efficient electrocatalysts for the hydrogen evolution reaction (HER). However, the large‐scale commercialization of Pt‐based catalysts suffers from the high cost. Therefore, ultralow‐Pt‐loading electrocatalysts, which can reach the balance of low cost and high HER performance, have attracted much attention. In this review, representative promising synthetic strategies, including wet chemistry, annealing, electrochemistry, photochemistry, and atomic layer deposition are summarized. Further, the interaction between different electrocatalyst components (transition metals and their derivatives) and Pt is discussed. Notably, this interaction can effectively accelerate the kinetics of the HER, enhancing the catalytic activity. At last, current challenges and future perspectives are briefly discussed.

## Graphical abstract

Several synthesis strategies are reviewed for preparing ultralow‐Pt‐loading electrocatalysts in hydrogen evolution reaction (HER). Furthermore, the interaction between different catalyst components and Pt is discussed. The mechanisms of transition metals and their derivatives to accelerate the kinetics of HER and enhance the HER catalytic activity are summarized and future research directions are proposed.

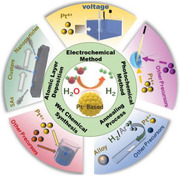

## 1 Introduction

Due to the rapid growth of the global population, and the perennial global warming, society is facing urgent energy crises and challenges.^[^ ^1^ ^]^ Thus, the pursuit of sustainable energy is rapidly growing. Meanwhile, renewable and fossil‐free fuels are being developed due to their environmental friendliness and abundance properties. The DOE (Department of Energy) and the IEA (International Energy Agency) have set targets for reducing the cost of hydrogen production with platinum (Pt)‐based electrocatalysts. The DOE has set a target of $40/kW for the cost of Pt‐based hydrogen production systems, while the IEA has set a target of $30/kW. These targets are aimed at making hydrogen a more competitive fuel source for a variety of applications, including transportation and energy storage. Amongst these, hydrogen molecules (H_2_) have the highest energy density (≈120 MJ kg^−1^) and carbon‐free character, making H_2_ to be widely recognized as one of the most promising alternatives in the next decade.^[^ ^2^ ^]^ According to the statistics, ≈95% of the industrial H_2_ generation relies heavily on the steam reforming, which not only requires a high consumption of fossil resources, but also releases carbon dioxide (CO_2_).^[^ ^3^, ^4^, ^5^ ^]^ Moreover, the demand for renewable energy stimulates the development of clean and efficient H_2_ production route. To this end, water electrolysis (2H_2_O(l)) → 2H_2_(g)+O_2_(g), Δ*G*_0_ = +273.2 kJ mol^−1^ and Δ*E*_0_ = 1.23 V versus normal hydrogen electrode (NHE), of which the cathode reaction potential is 0 V^[^ ^6^ ^]^ and anode reaction potential is 1.23 V^[^ ^7^, ^8^, ^9^ ^]^) has been regarded as one of the most encouraging alternatives to the steam reforming route.^[^ ^10^, ^11^ ^]^ The abundant water resources, high energy conversion rate, and nearly zero emissions of CO_2_ contribute to make water electrolysis feasible.^[^ ^12^ ^]^

Theoretically, water electrolysis involves two half‐reactions, hydrogen evolution reaction (HER) and oxygen evolution reaction (OER) on cathode and anode of an electrochemical device.^[^ ^10^, ^13^ ^]^ During the actual reaction process, the electrochemical reactions on the relevant electrodes are accompanied by an operating voltage (*E*_op_) over the theoretical value, which is called overpotential. The reason behind is that the *E*_op_ needs to overcome the activation energy of the theoretical overpotential (cathode: *E*_cathode_, anode: *E*_anode_) and the resistance of the internal electrolytic device (*E*_other_), as shown in Equation (1)

(1) $$E_{\mathrm{op}}=1.23\;V+E_{\mathrm{cathode}}+E_{\mathrm{anode}}+E_{\mathrm{other}}$$

**Figure 1:**
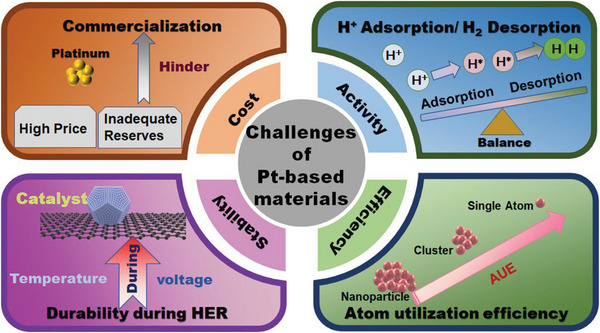
Main challenges for Pt‐based materials.

Hence, efficient water splitting needs of appropriate efficient electrocatalysts to diminish overpotentials for both HER and OER. To date, Pt‐based materials and their derivatives are generally regarded as the state‐of‐the‐art catalyst for HER,^[^ ^14^, ^15^, ^16^, ^17^, ^18^, ^19^ ^]^ owing to the outstanding hydrogen binding energy (the optimum adsorption and desorption capacity with hydrogen intermediates), near‐zero overpotentials, remarkable exchange current densities (*j*_0_), and small Tafel slopes.^[^ ^20^, ^21^ ^]^ However, there are still four main challenges (**Figure** **1**) which limit the commercialization of Pt‐based materials. The high cost and inadequate reserves limit the large‐scale usage of Pt‐based electrocatalysts.^[^ ^22^, ^23^, ^24^, ^25^, ^26^ ^]^ Thus, a pivotal challenge for HER is how to decrease the cost of Pt required in the electrocatalysts, which requires a significant improvement in Pt mass activity (MA, the catalytic current per unit mass of Pt).^[^ ^24^, ^27^, ^28^, ^29^ ^]^ As it is well‐known that the MA is strongly connected with the amount of exposed active sites, plentiful research effort has been devoted to downsizing Pt‐based nanoparticles to clusters or single atom (SA) level.^[^ ^30^, ^31^, ^32^, ^33^ ^]^ It is generally recognized that downsizing catalysts will create higher surface/volume ratio. Thereby, the isolated Pt sites can easily react with hydrogen intermediates, exhibiting good HER activity and improving the atom utilization efficiency (AUE).^[^ ^34^, ^35^, ^36^, ^37^ ^]^ Nonetheless, it is still challenging to maintain the stability of Pt‐based nanoparticles, because of the low‐resistance toward Ostwald ripening (the aggregation of downsized Pt‐based nanoparticles under a certain temperature) or an insufficient durability under a given voltage.^[^ ^38^, ^39^ ^]^ To overcome this, one strategy has been proposed to maintain stability of Pt‐based electrocatalysts during the electrocatalytic processes by loading nanoparticles onto supports,^[^ ^40^, ^41^, ^42^ ^]^ including carbon^[^ ^43^ ^]^ and metal‐based^[^ ^44^ ^]^ supports. These are accepted as promising supports for the electrocatalytic process because of their tunable electrical conductivity,^[^ ^45^, ^46^ ^]^ abundant defects,^[^ ^47^, ^48^ ^]^ and ability to undergo further chemical and structural modifications.^[^ ^49^, ^50^ ^]^ In addition, directly optimizing the intrinsic activity of every single active site is another feasible method to reduce the Pt usage.^[^ ^51^, ^52^, ^53^ ^]^ In this aspect, alloys and polymetallic compounds consisting of Pt and non‐noble metals (such as Co,^[^ ^54^, ^55^ ^]^ Ni,^[^ ^56^, ^57^, ^58^ ^]^ Fe,^[^ ^59^, ^60^ ^]^ Cu^[^ ^61^, ^62^ ^]^) have been broadly explored. Apart from the size effect of Pt, non‐noble metals can reconstruct the surface electronic structure through interactions.^[^ ^63^, ^64^, ^65^ ^]^ Furthermore, electronic modification can reduce the adsorption and desorption energy barriers of hydrogen intermediates.^[^ ^66^, ^67^, ^68^ ^]^

So far, much effort has been devoted to efficient hydrogen generation.^[^ ^69^, ^70^, ^71^, ^72^ ^]^ Most reports ^[^ ^43^, ^44^, ^73^, ^74^, ^75^ ^]^ discuss the production of Pt nanoparticles, Pt‐based alloys and Pt‐based heterogeneous electrocatalysts. Chen and his team summarized metal alloy electrocatalysts,^[^ ^75^ ^]^ especially introduced and analyzed the mechanism and application of Pt‐based alloy catalyst in hydrogen evolution reaction, discussed the progress in the design, preparation, and application of Pt‐based alloy electrocatalysts. In Xu's review,^[^ ^70^ ^]^ the principles of the HER under alkaline conditions are first introduced, followed by a discussion of the latest advances in Pt‐based heterostructured catalysts. Special focus is placed on approaches for enhancing the reaction rate by accelerating the Volmer step. Xu also provided the design principles for the future development of heterostructured nano‐ or microsized electrocatalysts. As discussed in Baek's review,^[^ ^74^ ^]^ Pt and other noble metals (Ru and Ir) are currently considered the most active materials for HER. Baek and his team mainly focus on the recent advances in noble‐metal‐based HER electrocatalysts. In particular, the synthesis strategies to enhance cost‐effectiveness and the catalytic activity for HER are highlighted. Nonetheless, large‐scale commercialization requires further reducing the cost of Pt‐based electrocatalysts and maximizing the AUE of Pt. Therefore, a viable project is to develop ultralow‐Pt‐loading electrocatalysts (UPLEs), which can realize a balance between the cost and HER activities. Comparing with the published reviews, this review mainly aims at providing a highly generalized summary about HER and how to synthesis the UPLEs through a simple strategy, such as (electrochemical method, photochemical method, annealing method, wet chemical method, atomic layer deposition (ALD) method). Moreover, the interaction between Pt and other components is discussed to further shed light on the charge modification on UPLEs. Although, UPLEs, Pt nanoparticles, Pt‐based alloys, and Pt‐based heterogeneous electrocatalysts are all materials used in electrocatalysis, which involves the use of an electrical current to catalyze a chemical reaction.^[^ ^76^ ^]^ However, they differ in their composition, structure, and properties. UPLEs are typically made up of Pt atoms dispersed on the supporters, which helps to reduce the amount of platinum needed while still maintaining high catalytic activity.^[^ ^77^ ^]^ These electrocatalysts typically have very low Pt loading, with a weight percentage of Pt less than 10 wt% and size less than 5 nm. Pt nanoparticles exhibit the size around 10 nm. They are typically supported on carbon or other materials to improve their stability and dispersion.^[^ ^78^, ^79^ ^]^ Pt nanoparticles are widely used in fuel cells and other electrochemical devices because of their high catalytic activity and stability.^[^ ^80^ ^]^ Pt‐based alloys are made by mixing Pt with other metals, such as nickel, cobalt, or palladium. These alloys often have improved properties over pure Pt, such as higher catalytic activity, better durability, and lower cost. Pt‐Ni and Pt‐Co alloys are commonly used in fuel cells and other electrochemical devices.^[^ ^81^ ^]^ Pt‐based heterogeneous electrocatalysts are materials where platinum is anchored onto a support material, such as metal oxides or other conductive materials.^[^ ^82^ ^]^ These materials are designed to improve the durability and stability of the catalyst, while also providing a high surface area for catalysis. Pt‐based heterogeneous electrocatalysts can be used in a wide range of electrochemical applications, including fuel cells, electrolysis, and electrochemical sensors. In summary, UPLEs use a minimal amount of platinum to achieve high catalytic activity, which can be designed as Pt nanoparticles, Pt‐based alloys and Pt‐based heterogeneous electrocatalysts. According to the recent published works,^[^ ^83^, ^84^, ^85^, ^86^ ^]^ as the commercialized 20%Pt/C is commonly used as the benchmark catalyst for HER. The Pt‐loading on the working electrode will be counted for 0.04 mg cm^−2^ as a strategy for blending 2 mg (catalyst powder) with 20 µL Nafion solution (5 wt%) and 980 µL mixture of isopropanol/ethanol/water. The definition of UPLEs should be the catalysts with less than 0.02 mg cm^−2^ Pt‐loading on the electrode. Otherwise, the Pt‐loading of catalysts should be lower than 10 wt% tested by inductively coupled plasma mass spectrometer (ICP‐MS).

Currently, the research progress of UPLEs involves the enhancement of the intrinsic activity by manipulating the morphology, composition, and construction of platinum‐based electrocatalysts. To shed some light on the interaction of Pt with other components, density functional theory (DFT) calculations are generally conducted. Although numerous catalysts have shown the potential to replace commercial Pt/C catalysts,^[^ ^87^, ^88^, ^89^, ^90^, ^91^, ^92^, ^93^, ^94^, ^95^, ^96^ ^]^ an integrated review about design and application of UPLEs is still needed to guide future research. Here, HER mechanisms and general evaluation measurements of HER are first introduced. Then, several typical synthetic methods of UPLEs and their HER performances are discussed. In the end, the interaction between different components of UPLEs is summarized. This review can provide a guidance to design and optimize Pt‐based electrocatalysts with excellent HER activity and competitive cost.

## 2 Fundamentals of HER

### 2.1 HER Mechanism

**Figure 2:**
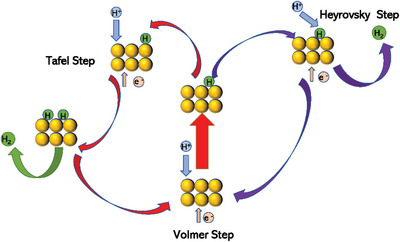
Different pathways of hydrogen evolution on the surface of electrocatalysts (blue represents proton, green represents hydrogen atom intermediate and hydrogen molecules, yellow represents metal atoms).

Based on several studies,^[^ ^97^, ^98^, ^99^ ^]^ the HER process can follow two different pathways: Volmer–Tafel and Volmer–Heyrovsky, as shown by **Figure** **2** and Equation (2)–(4)

(2) $$\mathrm{Volmer}\;\mathrm{step}:{\;H}^{+}+e^{-}\to H^{*}$$

(3) $$\mathrm{Tafel}\;\mathrm{step}:\;2H^{*}\to H_{2}$$

(4) $$\mathrm{Heyrovsky}\;\mathrm{step}:{\;H}^{*}+H^{+}+e^{+}\to H_{2}$$

In the HER process, the Volmer step is first performed. One proton (H^+^) in the electrolyte is adsorbed on the catalyst surface to form a hydrogen atom intermediate (H*), where * represents the active site on the catalyst surface.^[^ ^99^ ^]^ The Volmer step is also referred to as the discharge step according to Equation (2). Subsequently, the reaction pathways of HER are divided into two types. The Tafel step (desorption step, Equation (3)) generates hydrogen molecules by combining two adsorbed hydrogen atom intermediates, while the hydrogen molecules, generated by Heyrovsky step (electrochemical desorption step, Equation (4)), consist of an adsorbed hydrogen atom intermediate, a proton and an electron. It is obvious that H* plays a crucial role in the hydrogen generation. Thus, the kinetics of HER are influenced remarkably by the Δ*G*_H*_ of adsorption of H^*^.^[^ ^100^ ^]^ If Δ*G*_H*_ is too high to overcome, it is difficult for H^+^ to be adsorbed to the active sites on the catalyst surface, then the Volmer step will limit the rate of the HER. On the contrary, when ΔG_H_ is too low, it will lead to a high‐strength bonding among H* and the active sites, then H* will be tough to desorb, and the Tafel or Heyrovsky step will impose restrictions on the HER. In fact, there are three types of electrolytes in practical water electrolysis devices: acid, alkaline, and neutral solutions, which could lead to the different existence of H^+^ and the corresponding reaction step.^[^ ^96^ ^]^ For instance, in acidic electrolytes, H^+^ exists in the form of H_3_O^+^, while the concentration of H^+^ is low in the alkaline or neutral electrolytes, which leads to different reaction pathways depending on the electrolyte, as shown in **Table** **1**.

**Table 1:** Pathways of HER in different electrolyte media

Pathway	Acid	Alkaline or neutral
Volmer	H_3_O^+^+e^−^→H*+H_2_O	H_2_O+e^−^→H*+OH^−^
Tafel	2H*→H_2_	2H*→H_2_
Heyrovsky	H*+H^+^+e^−^→H_2_	H*+H_2_O+e^−^→H_2_+OH^−^

### 2.2 HER Evaluating Measurements

#### 2.2.1 Overpotential

**Figure 3:**
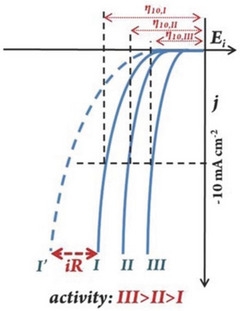
Schematic HER polarization curves on different electrocatalysts with iR correction and overpotentials indicated. Reproduced with permission.^[^ ^102^ ^]^ Copyright 2019, Wiley‐VCH.

The overpotential has been widely regarded as one of the most important measurements for assessing the activity of HER catalysts. Referring to the Nernst equation, under standard conditions, the potential of the HER relative to an ordinary hydrogen NHE is zero. However, the practical process requires an imposed potential to overcome some unfavorable problems, such as the kinetic energy barrier caused by high Δ*G*_H_ and resistance of electrolyte in three electrode measurement system.^[^ ^4^, ^101^ ^]^ Commonly, the corrected overpotential (*E*_c_) should be measured after the iR compensation (Equation (5)). Also, the current density at 10 mA cm^−2^ equals to the 12.3% device efficiency for solar water‐splitting, so the overpotential at 10 mA cm^−2^ is normally noted to evaluate the HER activity of the electrocatalysts,^[^ ^102^ ^]^ as shown in **Figure** **3**

(5) $$E_{c}=E_{\mathrm{measured}}-\mathrm{Ir}$$

#### 2.2.2 Tafel Slope

**Figure 4:**
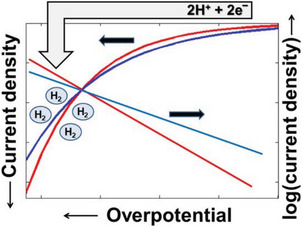
The relationship among Tafel slope, current density, and overpotential. Reproduced with permission.^[^ ^103^ ^]^ Copyright 2018, American Chemistry Society.

The Tafel slope (*b*), relating to the rate of HER process, represents the intrinsic activity of an electrocatalyst. In Equation (6), *E*_op_ is plotted as the function of log(*j*/*j*_0_), in which *j* is the current density and *j*_0_ is the exchange current density. Meanwhile, *b* can be valued from the linear extended part of Tafel slope (**Figure** **4**).^[^ ^103^ ^]^ The *j*_0_ is also an important kinetic parameter to describe the intrinsic catalytic activity of electrocatalysts under a reversible environment, and *j*_0_ can be determined when *E*_op_ is valued as Zero.^[^ ^104^ ^]^ The kinetics of the electron transfer will be accelerated when *b* decreases at the same current density. Efficient HER electrocatalysts should deliver both large *j*_0_ and small values of *b*.

In general, we can infer the reaction path of the HER process from the Tafel slope obtained from electrochemical tests. If the Tafel slope is close to 30 mV dec^−1^, this indicates that the HER proceeds through the Volmer–Tafel step, which is the rate‐limiting step; if the Tafel slope is close to 40 mV dec^−1^, this indicates that the HER proceeds through the Volmer–Heyrovsky step, Heyrovsky is the rate‐limiting step. Both cases indicate that the adsorption of H* on the catalyst surface is easy to proceed, and the reaction rate of HER is mainly affected by the desorption of H*.^[^ ^105^ ^]^ When the Tafel slope reaches 120 mV dec^−1^, the Volmer step will become the rate‐limiting step, which indicates that H^+^ is not easily adsorbed to the active sites on the catalyst surface^[^ ^104^ ^]^

(6) $$E_{\mathrm{op}}=b\log\left(j/j_{0}\right)$$

#### 2.2.3 Electrochemical Impedance Spectroscopy (EIS)

**Figure 5:**
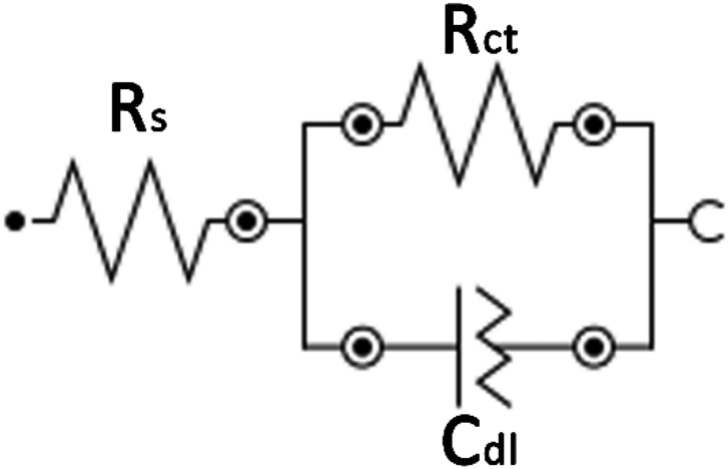
EIS equivalent model of the Faradaic impedance of HER.

Both Volmer–Tafel and Volmer–Heyrovsky include the adsorption of H* on the electrode surface. The rate of this process can be evaluated by using the electrochemical measurement system.^[^ ^106^ ^]^ The *R*_s_ (internal resistance of the electrode and electrolyte) and *R*_ct_ (charge transfer resistance of electrocatalysts) can be measured by EIS Nyquist model (**Figure** **5**). Theoretically *R*_s_ is generated because the electrolyte mainly relies on ionic conduction, and when an electric field is applied, the movement speed of ions in the electrolyte becomes faster, thus the resistance decreases. Since HER is always measured within a narrowed potential range, *R*_s_ will not change that much in the same electrochemical measurement system. So *R*_ct_ can be directly related to the rate of the charge transfer process at the interface of electrocatalysts. As for the hydrogen evolution process, the smaller *R*_ct_, the faster the charge transfer rate and lower overpotential.

#### 2.2.4 Durability

Durability is another key measurement for evaluating the commercialization potential of HER catalysts. Currently, there are two main methods for durability test: voltammetry (cyclic voltammetry (CV) or linear sweep voltammetry (LSV)) and chronoamperometry.^[^ ^6^, ^34^ ^]^ Both CV and LSV results compare the variation in the overpotential before and after a cyclic test (≥1000 cycles in the range including onset‐potential and overpotential at 10 mA cm^−2^. A small variation after cyclic tests is allowed, which manifests the stable HER activity of electrocatalysts. The chronoamperometry is used to observe current density change of the electrocatalyst, the potential applied to this method is exactly corresponding to the current density at 10 mA cm^−2^. The durability test should last not be less than 12 h. The longer test means the better durability.

#### 2.2.5 Turnover Frequency

In the HER process, the turnover frequency (TOF) is the number of hydrogen molecules generated by per active site per second.^[^ ^107^ ^]^ Therefore, TOF can also evaluate the intrinsic activity and efficiency of each active site. Recently, TOF for HER electrocatalysts can be calculated based on Equation (6), where *I* represents the current (*A*), *F* represents the Faraday constant (96 485.3 C mol^−1^), *x* represents the number of electron transfer during the HER process, and n represents the number of moles of the active sites^[^ ^87^ ^]^

(7) $$\mathrm{TOF}=\frac{I}{xnF}$$

However, as for some of the latest HER catalysts, especially heterogeneous materials, the TOF value is inaccurate due to the difficulty in obtaining the precise number of moles of surface catalytic sites. Nevertheless, TOF still provides a feasible way to compare the activities of HER catalysts under the same or similar systems.

## 3 Synthesis and Design of UPLEs

Many efforts have provided reliable strategies to prepare high‐performance UPLEs by regulating the size, composition, and structure of platinum‐based active sites. In this regard, several main strategies for synthesizing UPLEs have been introduced, including electrochemical and photochemical reduction reactions, high‐temperature annealing, wet chemistry method, and ALD, etc.

### 3.1 Electrochemical Method

The electrochemical method refers to a process in which an electrochemical cell is used to synthesize or modify materials by applying an electric potential difference across an electrolytic solution containing the precursor material. The specific details of the electrochemical method can vary depending on the specific application and materials being used. Thus, electrochemical methods are gradually recognized as a simple, low energy consumption, and environmental‐friendly strategy to prepare UPLEs.^[^ ^108^ ^]^ In addition, the amount and size of Pt‐based materials can be accurately controlled through electroplating parameters, and its outer layer can be modified to improve the AUE of Pt. There are two different types of electrochemical methods: electrochemical etching and electrochemical reduction. Commonly used electrolytes in electrochemical methods include aqueous solutions of acids or salts, such as sulfuric acid or sodium chloride. However, nonaqueous electrolytes, such as organic solvents, can also be used for specific application. The Pt precursor used in electrochemical methods can also vary depending on the desired application. Common Pt precursors include H_2_PtCl_6_, Pt(acac)_2_ and Pt counter electrode (such as Pt foil, etc.), which can be reduced to form Pt nanoparticles or thin films on a substrate. The corresponding potential range used in electrochemical methods can also vary depending on the specific application and materials being used. Generally, the potential range will be chosen to allow for the reduction or oxidation of the precursor material, while avoiding unwanted side reactions. For example, the reduction of Pt^2+^ typically occurs in a potential range of −0.4 to −1.0 V versus a standard hydrogen electrode (SHE).

**Figure 6:**
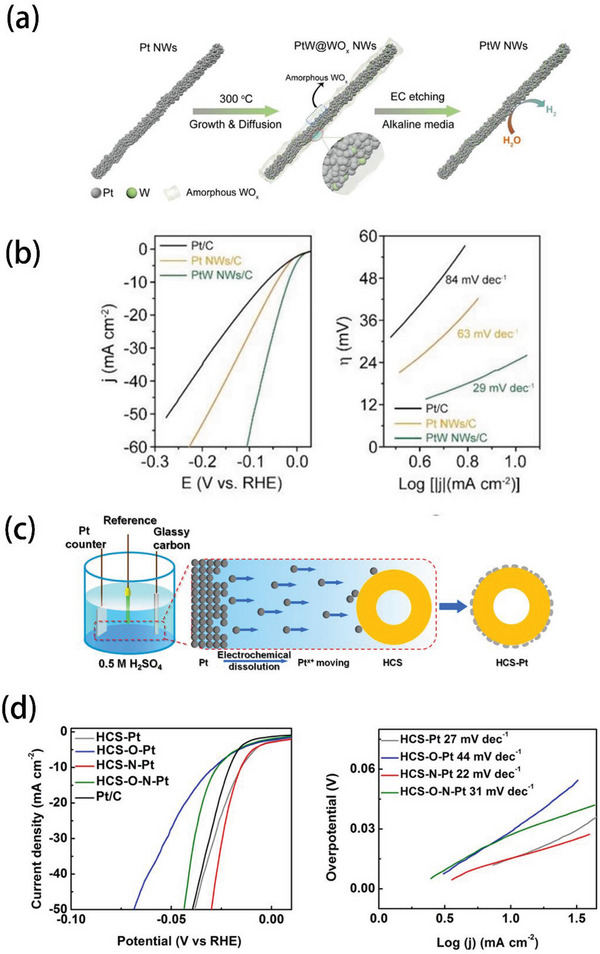
a) Preparation process of PtW NWs/C. b) Corresponding HER performance and Tafel slope. Reproduced with permission.^[^ ^109^ ^]^ Copyright 2022, Wiley‐VCH. c) Preparation process of HCS‐N‐Pt. d) Corresponding HER performance and Tafel slope. Reproduced with permission.^[^ ^111^ ^]^ Copyright 2018, American Chemical Society.

As shown in **Figure** **6**a, Huang et al. obtained a PtW NWs/C catalyst through a electrochemical (EC) etching,^[^ ^109^ ^]^ by the LSV process at a scan rate of 5 mV s^−1^ in 1.0 m KOH for seven cycles with Pt(acac)_2_, CATB, W(CO)_6_ in an oleyl‐amine solution as reactants to synthesize PtW@WO*_x_* NWs/C . The as‐synthesized PtW NWs/C catalyst exhibited an extremely low overpotential of 18 mV at 10 mA cm^−2^ in 1.0 m KOH, and its Tafel slope was 29 mV dec^−1^, reflecting that the current density of PtW NWs/C catalyst increased faster with the potential rising than commercial Pt/C catalysts (Figure 6b). In addition, it has been reported that Pt can be dissolved from the Pt counter electrode (such as Pt foil, etc.) and reduced onto the working electrode during the HER test. The dissolution and reduction processes are more severe in acid electrolysis than in alkaline conditions, which significantly affect the accuracy of the measured HER activity.^[^ ^110^ ^]^ Thus, Pt cations can be easily reduced on the cathode in the electrolyte during the dissolution process. Owing to this strategy, Liu and his group utilized the Pt counter electrode to replace the normal Pt precursor, and successfully prepared nanostructured Pt clusters on nitrogen‐functionalized hollow carbon sphere (HCS‐N) with an ultralow Pt loading (1.7 µg cm^−2^ and 0.05 wt%),^[^ ^111^ ^]^ as shown in Figure 6c. The HCS‐N‐Pt catalyst showed the best HER activity compared to other HCS‐O‐Pt within this report, HCS‐O‐N‐Pt and commercial Pt/C catalysts, only requiring an overpotential of 14.4 mV to reach 10 mA cm^−2^ and the Tafel slope of 22 mV dec^−1^ in 0.5 m H_2_SO_4_ (Figure 6d).

However, some disadvantages of electrochemical method hamper the large‐scale application, including 1) electrode stability: electrodes can be damaged or dissolved during electrochemical reactions, which can limit the lifetime of the equipment and require frequent maintenance. 2) cost: electrochemical methods can be expensive due to the need for specialized equipment and materials. 3) complexity: electrochemical methods can be complexed and required skilled operators to ensure the reaction proceeds correctly. Thus, we provide improvement strategies to overcome the shortcomings. First, developing more stable electrode materials can improve the lifetime of electrochemical equipment and reduce maintenance costs. Second, automating electrochemical processes can reduce the need for skilled operators and improve consistency and reproducibility. Also, developing scalable electrochemical processes can reduce costs and increase efficiency, making electrochemical methods more competitive with traditional methods. Furthermore, using renewable energy sources to power electrochemical processes can further reduce the environmental impact of electrochemical methods.

### 3.2 Photochemical Method

Photochemical reduction, including the processes of nucleation, growth, and conversion to metal nanoparticles, has been widely used to synthesize noble metal nanoparticles.^[^ ^78^, ^112^, ^113^, ^114^ ^]^ Uniformly dispersed metal clusters or single atoms can be prepared by reducing the rate of reaction nucleation. At the same time, the use of low‐temperature light sources such as ultraviolet rays can effectively prevent the aggregation of metal atoms. Meanwhile, low‐temperature solvents can also be used to increase the energy barrier of the nucleation and achieve inhibition. Photochemical reduction can also immobilize highly dispersed metal nanoparticles on carbon or metal oxide supports. The photochemical method is environmentally friendly, low energy requiring, and highly efficient.

**Figure 7:**
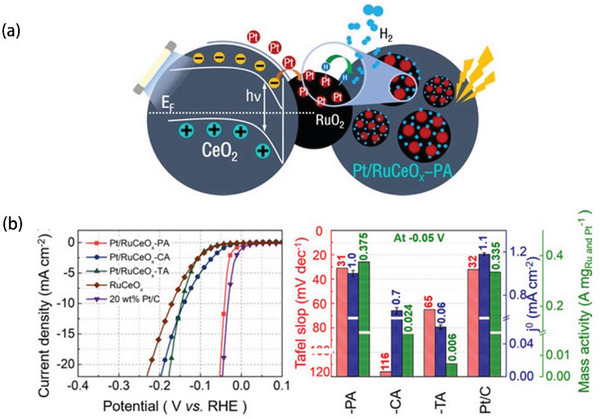
a) Preparation process of Pt/RuCeO*_x_*‐PA. b) Corresponding HER performance, Tafel slope, and mass activity. Reproduced with permission.^[^ ^112^ ^]^ Copyright 2020, Wiley‐VCH.

**Figure** **7**a illustrates the work by Wang et al., who diluted RuCeO*_x_* and H_2_PtCl_6_ solution into ultrapure water, then exposed the solvent to an ultraviolet‐lamp (50 W) for 3 h to obtain Pt/RuO*_x_*‐PA electrocatalyst (with only 0.49 wt% Pt loading).^[^ ^112^ ^]^ Figure 7a shows that under the ultraviolet light exposure, many photo‐induced electron‐hole pairs could be isolated from the RuO_2_/CeO_2_ interface, while photoelectrons transfer to the RuO_2_ surface to help anchoring Pt atoms. The HER performance of Pt/RuO*_x_*‐PA was evaluated in 0.5 m H_2_SO_4_ electrolyte. It is obvious that Pt/RuO*_x_*‐PA owned impressive HER activities (the overpotential of 41 mV at 10 mA cm^−2^ and the Tafel slope of 31 mV dec^−1^), which are comparable to those found for commercial Pt/C, and much better than other as‐prepared electrocatalysts (Figure 7b).

**Figure 8:**
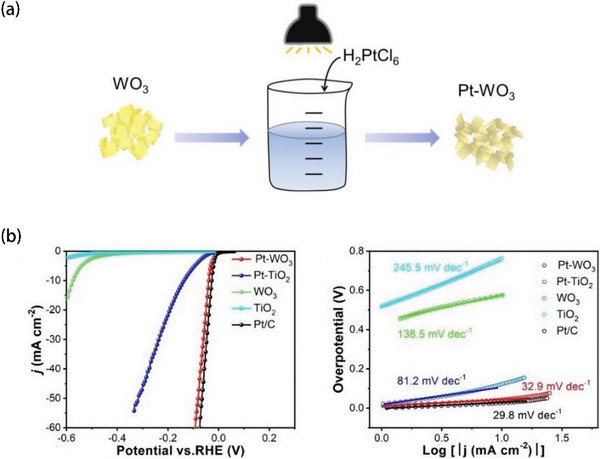
a) Preparation process of Pt‐WO_3_. b) Corresponding HER performance and Tafel slope. Reproduced with permission.^[^ ^113^ ^]^ Copyright 2020, Elsevier.

Additionally, Wang and coworkers mixed H_2_PtCl_6_ solution with pre‐prepared WO_3_ nanosheets, then the mixed solution was stirred under the simulated solar irradiation of a xenon lamp for 2 h (**Figure** **8**a). Finally, Pt‐WO_3_ (with 4.426 wt% Pt) was prepared after washing.^[^ ^113^ ^]^ As depicted in Figure 8b, Pt‐WO_3_ exhibited the outstanding HER activity (39 mV at 10 mA cm^−2^, 32.9 mV dec^−1^), remarkably close to the 20 wt% Pt loading commercial catalyst and higher than TiO_2_ based catalyst and Pt/C with the same Pt content.

However, there are still some limitations for this approach. Since most of the solvent used in the photochemical strategy is ultrapure water, materials cannot be used if they are unstable in ultrapure water. Moreover, Pt atoms do not have a very strong respond to light irradiation. Therefore, photochemical reduction requests that carriers should be photo‐responsive and rich in defects or heteroatoms to strongly anchor the Pt atoms.^[^ ^114^ ^]^

Several improvements of photochemical method are provided. On the one hand, the photochemical reduction of Pt precursor to Pt nanoparticles involves several reaction parameters such as the type of solvent, the concentration of the precursor, the type of reducing agent, and the intensity of light. An optimization of these parameters can lead to a higher yield of Pt nanoparticles with a narrower size distribution. On the other hand, surfactants are essential in controlling the size and stability of nanoparticles synthesized by the photochemical method. The choice of surfactant can significantly impact the size, shape, and dispersion of Pt nanoparticles. The use of suitable surfactants can improve the stability and size distribution of Pt nanoparticles.

### 3.3 Annealing Method

Annealing is a widely adopted strategy to synthesize single‐atom metal or alloy catalysts and can be conducted in a variety of atmospheres for the heat treatment, including argon (Ar), nitrogen (N_2_), hydrogen and nitrogen mixture (H_2_/N_2_). The metal precursor is decomposed at a certain temperature to obtain a single metal atom or nucleate and grow into an alloy. The influence of temperature on Pt nanoparticles and clusters can have several effects, including changes in their size, structure, and properties. As the temperature increases, the size and morphology of Pt nanoparticles and clusters can change. This can occur due to the thermal activation of surface atoms, which can lead to particle aggregation or coalescence. At higher temperatures, Pt nanoparticles may also undergo Ostwald ripening, where smaller particles dissolve and deposit onto larger particles, resulting in a shift in size distribution. Pt nanoparticles and clusters can exist in different crystal structures, such as face‐centered cubic (fcc), hexagonal close‐packed (hcp), and others. The crystal structure of Pt nanoparticles can change with temperature due to the thermal activation of surface atoms, which can lead to structural transformations from fcc to hcp or vice versa. Moreover, Pt nanoparticles and clusters can be subject to thermal degradation at high temperatures, which can lead to the loss of their catalytic activity or even structural disintegration. The thermal stability of Pt nanoparticles and clusters can be improved by modifying their size, morphology, and crystal structure. In addition, some supports are also obtained by high temperature pyrolysis,^[^ ^115^, ^116^, ^117^ ^]^ including graphene, metal–organic frameworks (MOFs) and some transition metal oxides. These carriers own strong electrical conductivity and are suitable for modification in different electrochemical systems. In addition, these materials have abundant specific surface area and porous structures, which are suitable for immobilizing and confining Pt ions.

**Figure 9:**
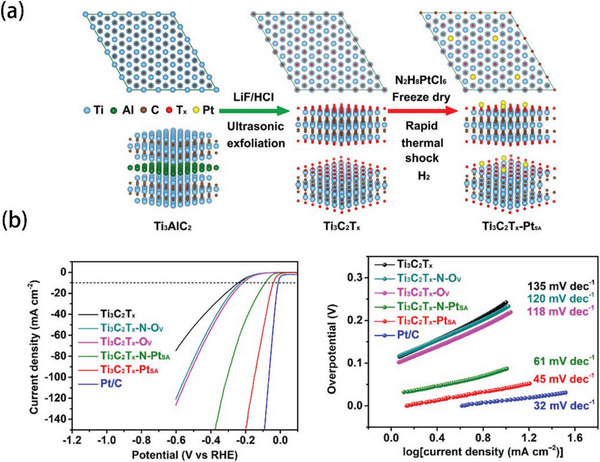
a) Preparation process of Ti_3_C_2_T*_x_*‐Pt_SA_. b) Corresponding HER performance and Tafel slope. Reproduced with permission.^[^ ^118^ ^]^ Copyright 2022, American Chemical Society.

As displayed in **Figure** **9**a, Gong and his team synthesized Pt SAs by carrying out a rapid thermal shock.^[^ ^118^ ^]^ In the first step, N_2_H_8_PtCl_6_ solution was mixed with the supporting material (Ti_3_C_2_T*_x_*). Second, the precursor was annealed at 400 °C for 10 min under a 10% H_2_/N_2_ atmosphere. As a result, the catalyst (Ti_3_C_2_T*_x_*‐Pt_SA_) with oxygen vacancies and highly dispersed Pt SAs (0.84 wt%) was obtained. As shown in LSV curves (Figure 9b), Ti_3_C_2_T*_x_*‐Pt_SA_ had an overpotential of 38 mV at 10 mA cm^−2^ in 0.5 m H_2_SO_4_, much lower than other non‐Pt‐based electrocatalysts and commercial Pt/C. Besides, Ti_3_C_2_T*_x_*‐Pt_SA_ showed the fastest HER kinetics with a Tafel slope of 45 mV dec^−1^.

**Figure 10:**
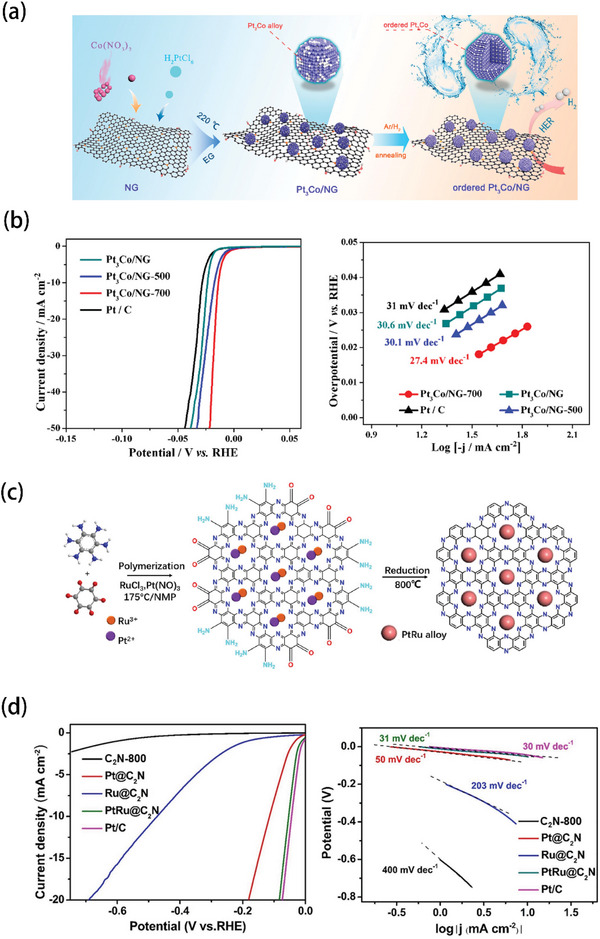
a) Preparation process of Pt_3_Co/NG‐700. b) Corresponding HER performance and Tafel slope. Reproduced with permission.^[^ ^119^ ^]^ Copyright 2020, American Chemical Society. c) Preparation process of PtRu@C_2_N. d) Corresponding HER activities in acid conditions. Reproduced with permission.^[^ ^120^ ^]^ Copyright 2022, Elsevier.

Cheng et al. prepared ultrafine and ordered Pt_3_Co alloy nanoparticles,^[^ ^119^ ^]^ as shown in **Figure** **10**a. The Pt_3_Co nanoparticles were first anchored onto the nitrogen‐doped graphene by ethylene glycol method (220 °C, 4 h). The Pt_3_Co/NG‐700 (9.6 wt% Pt loading) catalyst was synthesized by thermal treating at 700 °C under a 10% H_2_/N_2_ atmosphere. It is clear from Figure 10b that Pt_3_Co/NG‐700 catalyst exhibited remarkable HER performance, including lower overpotential (13 mV at 10 mA cm^−2^) and lower Tafel slope (27.4 mV dec^−1^) than Pt/C. In addition, another alloy catalyst (PtRu@C_2_N) was obtained by Fu et al.^[^ ^120^ ^]^ As shown in Figure 10c, the catalyst was prepared by annealing under 800 °C for 2 h under Ar atmosphere. Interestingly, Fu reported a metal‐support interaction between PtRu nanoparticles and C_2_N nanosheets, resulting in the improvement of HER activity. In 0.5 m H_2_SO_4_ electrolyte, PtRu@C_2_N exhibited an overpotential of 52 mV at 10 mA cm^–2^, with a small Tafel slope of 31 mV dec^–1^ (Figure 10d). This highly enhanced HER performance and fast kinetics can be attributed to the well‐designed electronic structure of PtRu nanoparticles and C_2_N nanosheets, which reduces the adsorption energy barrier of hydrogen intermediates.

Hence, proper design of the interface, regarding composition and density is critical for HER in a wide range of pH. In the next step, introducing components favorable for HER kinetics at the interface can further reduce the usage of Pt and improve the HER activity of the catalyst. The parameters of annealing and mechanism between different components will become the research focus.

### 3.4 Wet Chemical Method

Due to the prospect of commercial application, wet chemical methods have been widely used in the synthesis of Pt‐based materials. Wet chemical methods include galvanic replacement (GR),^[^ ^34^, ^121^ ^]^ impregnation,^[^ ^122^, ^123^ ^]^ chemical reduction (CR),^[^ ^87^, ^124^, ^125^ ^]^ etc.

**Figure 11:**
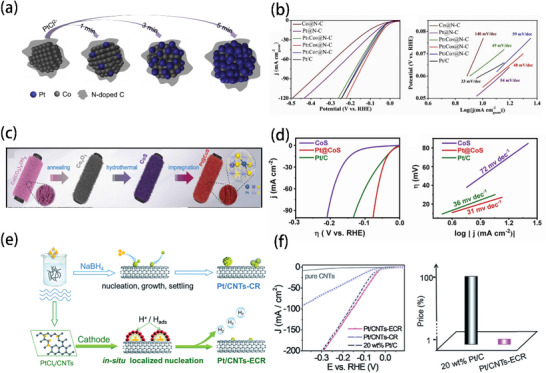
a) Preparation process of Pt_2_Co_8_@N‐C. b) Corresponding HER performance and Tafel slope. Reproduced with permission.^[^ ^121^ ^]^ Copyright 2019, Elsevier. c) Preparation process of Pt@CoS nanowires. d) Corresponding HER performance and Tafel slope. Reproduced with permission.^[^ ^122^ ^]^ Copyright 2022, Elsevier. e) Preparation process of Pt/CNTs‐ECR and Pt/CNTs‐CR. f) Corresponding HER performance. Reproduced with permission.^[^ ^124^ ^]^ Copyright 2019, Royal Society of Chemistry.

Cheng and his team successfully used the GR method to prepare Pt_2_Co_8_@N‐C, as shown in **Figure** **11**a.^[^ ^121^ ^]^ This method follows the reaction route of Co + PtCl_4_^2−^→ Co^2+^ + Pt + 4Cl^−^. In Figure 11b, the HER performance of fabricated Pt_2_Co_8_@N‐C catalyst was measured, a 47 mV overpotential at 20 mA cm^–2^ and a Tafel slope of 48 mV dec^–1^, were close to commercial Pt/C (53 mV and 33 mV dec^–1^). In addition, this work provided a simple and practicable mean for Pt‐based alloys. Wang and coworkers used the impregnation to synthesize Pt@CoS nanowires (Pt/Co atomic ratio of 0.85%) (Figure 11c).^[^ ^122^ ^]^ In an alkaline electrolyte (1 m KOH), Pt@CoS catalyst showed a superior catalytic performance of 28 mV overpotential at 10 mA cm^–2^ and a Tafel slope of 31 mV dec^–1^, much higher than other obtained catalysts and Pt/C (Figure 11d). In addition, combined with more details in this research, Pt atoms prefer to populate the Co position and form a four‐coordination structure during the impregnation process, which is similar to GR method. According to Wang's research (Figure 11e),^[^ ^124^ ^]^ they compared the CR method (by using NaBH_4_) with the in situ electrochemical method (ECR). They noticed that Pt nanoparticles (NPs) were easier to aggregate during the CR process, while ECR could synthesize ultrafine and separated Pt NPs. As shown in Figure 11f, the corresponding HER performance of Pt/CNTs‐ECR (0.3 wt% Pt loading) was higher, and the total cost was much lower than the commercial Pt/C. These studies demonstrated that although the wet chemical method has the advantage of being easy to operate, the control of catalytic sites still highly requires combining with the original metal sites or defects in the carriers.

However, several key differences between laboratory‐scale and industry‐scale production of wet chemical method hinder the commercialization. First, laboratory‐scale production of catalysts typically involves batch production, whereas industry‐scale production involves continuous production. In batch production, each reaction is carried out in a single vessel, whereas in continuous production, the reaction takes place in a series of interconnected vessels. This difference can affect the consistency of the product and the efficiency of the production process. Second, the scale of production is much larger in industry‐scale production, which means that the production process needs to be more robust and efficient. This requires specialized equipment and processes that can handle the large quantities of materials involved. Third, laboratory‐scale production is typically carried out under tightly controlled conditions, with precise measurements of all variables. In industry‐scale production, process control is more challenging, more convenient experimental conditions need to be proposed to ensure consistent quality and performance. Therefore, researchers are working hard to develop facile methods that can prepare high‐performance UPLEs.

### 3.5 ALD Method

ALD is known as one of the most advanced technologies for depositing atomic thin films onto the substrate. Moreover, ALD can be applied to obtain Pt SAs, owing to its precisely controllable deposition layers.^[^ ^16^, ^22^, ^126^ ^]^ Specifically, ALD is a thin film deposition technique used to create precise and conformal coatings on a substrate surface. Further, ALD is a self‐limiting and gas‐phase process where a substrate surface is exposed to alternate pulses of two or more precursors, resulting in the formation of a thin film on the surface. One of the main advantages of ALD is its ability to create precise and uniform coatings, even on complex geometries and high aspect ratio structures. Also, ALD is also capable of depositing a wide range of materials, including metals, oxides, nitrides, and sulfides, with precise control over thickness, composition, and morphology. Thus, ALD has become a popular technique for preparing single‐atom catalysts (SACs) due to its ability to control the size and distribution of metal atoms on a support material. SACs are a type of catalyst where individual metal atoms are dispersed on a support material, resulting in high catalytic activity and selectivity. The performance of SACs is highly dependent on the size and distribution of the metal atoms, making ALD an ideal technique for their preparation.

**Figure 12:**
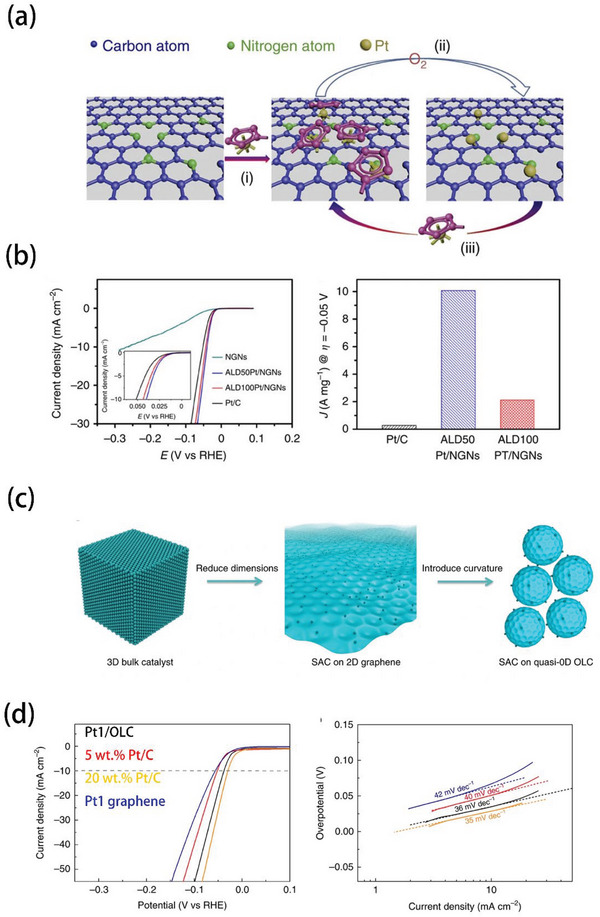
a) Preparation process of ALDPt/NGNs. b) Corresponding HER performance. Reproduced with permission.^[^ ^16^ ^]^ Copyright 2016, Springer Nature. c) Preparation process of Pt1/OLC. d) Corresponding HER performance and Tafel slop. Reproduced with permission.^[^ ^22^ ^]^ Copyright 2019, Springer Nature.

As displayed in **Figure** **12**a, Pt single atoms catalyst (ALDPt/NGNs) were deposited on N doped graphene nanosheets by using MeCpPtMe_3_ and oxygen as precursors through an ALD method.^[^ ^16^ ^]^ During the process, the N‐dopant influenced the reaction between MeCpPtMe_3_ and NGNs, and this chemical bond contributed to form anchored Pt SAs. Sun et al. also controlled the Pt loading with an ALD program, the catalyst with only 2.1 wt% Pt loading was obtained after 50 cycles. Notably, the HER catalytic activity of ALDPt/NGNs decreased with the increasing ALD cycle number (Figure 12b). These results suggested that downsizing Pt nanoparticles to clusters or SA levels can improve their AUE and minimize the cost. Song et al. used ALD to deposit Pt SAs onto onion‐like carbon nanospheres (OLC), as shown in Figure 12c.^[^ ^22^ ^]^ The surface‐oxidized detonation nanodiamonds (DNDs) were treated under various temperatures to obtain OLC to precisely adjust the oxygen species and defects. The engineered vacancy defects and functional groups on the OLC were aimed to stabilize Pt SAs. Besides, OLC owned the high curvature leaded to accumulation of electrons around Pt regions, which could accelerate HER kinetics. Consequently, Pt1/OLC (0.27 wt% Pt content, “1” means a single ALD cycle process) achieved an overpotential of 38 mV at 10 mA cm^−2^ and a Tafel slope of 36 mV dec^−1^, which were comparable to commercial Pt/C and much higher than Pt SAs on graphene (Figure 12d).

However, there are also some disadvantages to ALD, such as running costs and technical challenges. One major disadvantage of ALD is the high cost of equipment and materials. ALD requires specialized equipment and materials, such as high‐purity precursors and sophisticated reaction chambers, which can be expensive to purchase and maintain. Additionally, the process can be slow and requires multiple cycles, which can increase the overall cost of production. Another challenge with ALD is that it can be technically complex and difficult to optimize. ALD requires precise control over reaction conditions, such as temperature, pressure, and gas flow rates, which can be challenging to achieve and maintain. The process also requires careful monitoring and control of precursor delivery and surface reactions, which can be difficult to achieve with some materials. Despite these challenges, ALD can be combined with other traditional synthesis strategies to overcome some of these limitations. For example, ALD can be used in combination with physical vapor deposition (PVD) to create composite coatings with improved properties to create complex multilayer structures with precise control over thickness and composition.

In conclusion, various methods can be applied to synthesize UPLEs. However, they all have advantages and limitations. In the process of synthesizing UPLEs, we should pay more attention to the design of the carrier, including manufacturing defects, oxygen vacancies, introduction of functional groups, etc. The preparation method of UPLEs (ultrafine particles of transition metal oxides or mixed metal oxides) plays a crucial role in determining their morphologies, structures, growth modes, chemical bonding strength, particle size distribution, and catalytic activity. In this response, we will discuss each of these areas in more detail. Various preparation methods can yield different morphologies and structures of UPLEs. For example, electrochemical method, photochemical method, and wet chemical methods can produce porous and amorphous structures, while hydrothermal synthesis can lead to well‐defined nanocrystals. The morphology and structure of UPLEs can significantly affect their properties, such as surface area, pore size, and surface chemistry, which in turn influence their performance in catalysis and other applications. On the other hand, the growth mode of UPLEs during preparation can also affect their properties. Different preparation methods can result in different growth modes, such as particle growth by aggregation or nucleation and growth. For example, the annealing method can lead to rapid nucleation and growth, resulting in small particle size and high surface area, while solvothermal synthesis (one of the wet chemical methods) can lead to slow nucleation and growth, resulting in larger particle size and lower surface area. The preparation method can also affect the chemical bonding strength of UPLEs. For instance, calcination of UPLEs at high temperatures can lead to the formation of stronger metal‐oxygen bonds, while the use of surfactants can lead to weaker bonds due to the interaction of surfactants with metal ions during synthesis. The particle size and distribution of UPLEs are also influenced by the preparation method. For example, precipitation methods can lead to large particle size and broad size distribution, while hydrothermal synthesis can produce uniform particle size distribution. Further, the catalytic activity of UPLEs can also be affected by the preparation method. For example, the use of different surfactants during synthesis can affect the surface chemistry of UPLEs, which in turn influences their catalytic activity. Similarly, the size and morphology of UPLEs can affect their catalytic activity by affecting their surface area and the number of active sites. In summary, the preparation method of UPLEs can significantly affect their morphology, structure, growth mode, chemical bonding strength, particle size distribution, and catalytic activity. Researchers should carefully consider these factors when selecting a preparation method for UPLEs and when interpreting their properties and performance in applications.

The strategies mentioned above are only tools for experimental design, and they can be used alone or in combination. In recent years, UPLEs have been the trend of HER catalysts, because of their outstanding catalytic performance (**Table** **2**). UPLEs can reach the current density of 10–20 mA cm^−2^ with only a very small voltage both in 0.5 m H_2_SO_4_ and 1.0 KOH electrolyte. Moreover, the addition of Pd and Mo has been shown to improve the HER performance of UPLEs under high current densities, achieving the durability over 100 h, as they can alter the electronic and surface properties of the catalyst. In conclusion, while UPLEs remain the most efficient electrocatalysts for HER, their performance under high current density conditions can be improved by carefully designing the catalyst structure and composition to minimize the bubble effect and enhance mass transfer.

**Table 2:** Recently reported Pt‐based HER catalysts

Catalyst	Overpotential [mV]	Current density [mA cm^−2^]	Tafel slope [mV dec^−1^]	Pt loading	Electrolyte	Refs.
1.2%PtCo/NPC	14.2	10	21.2	1.2 wt%	0.5 m H2SO4	[34]
Pt‐T/G‐150	30	10	30	1.42 wt%	0.5 m H2SO4	[127]
Pt_1_/OLC	38	10	36	0.27 wt%	0.5 m H2SO4	[22]
Pd‐Cu/Pt	22.8	10	25	—	0.5 m H2SO4	[62]
PtCu NSs/C	26.8	10	28.4	—	0.5 m H2SO4	[23]
Pt/RuCeO*_x_*‐PA	41	10	31	0.49 wt%	0.5 m H2SO4	[112]
Pt/GNs	25	10	33	14.7 wt%	0.5 m H2SO4	[128]
Pt/NPC	21.7	20	36.3	1.82 wt%	0.5 m H2SO4	[129]
Pt/def‐WO_3_@CFC	42	10	61	—	0.5 m H2SO4	[89]
Ni‐MOF@Pt	43	10	30	20 wt%	0.5 m H2SO4	[130]
	102	10	88		1.0 KOH	
Pt‐TiN NTAs	71	10	46.4	—	0.5 m H2SO4	[131]
ALD50Pt/NGNs	—	10	29	2.1 wt%	0.5 m H2SO4	[16]
Pt_2_Co_8_@N‐C	47	20	48	—	0.5 m H2SO4	[121]
Pt@CoS	28	10	31	—	1.0 m KOH	[122]
Pt_3_Co/NG‐700	13	10	27.4	9.6 wt%	0.5 m H2SO4	[119]
Ti_3_C_2_T*_x_*‐Pt_SA_	38	10	45	0.84 wt%	0.5 m H2SO4	[117]
Pt_3_Ni_3_ NWs/C‐air	40	10	—	—	1.0 m KOH	[132]
Pt/RuO*_x_*‐PA	41	10	31	0.49 wt%	0.5 m H2SO4	[112]
Pt‐WO_3_	39	10	32.9	4.43 wt%	0.5 m H2SO4	[113]
HCS‐N‐Pt	14.4	10	22	0.05 wt%	0.5 m H2SO4	[111]
PtW NWs/C	18	10	29	—	1.0 m KOH	[109]
Pt@mh‐3D MXene	12	10	24.2	2.4 wt%	0.5 m H2SO4	[133]
	31	10	41		1.0 m KOH	
Pt/HMCS	20.7	10	28.3	5.08 wt%	0.5 m H2SO4	[134]
	46.2	10	48.1		1.0 m KOH	
Pt‐Ni(N)	13	10	29	—	1.0 m KOH	[96]
Pt‐Co_2_P	2	10	44	3.86 wt%	1.0 m KOH	[66]
	58	100	—		1.0 m KOH	
Pt‐Pd@NPA	28.1	10	31.2	4.9 µg cm^−2^	0.5 m H2SO4	[135]
	107.5	500	—		0.5 m H2SO4	
	137.7	1000	—		0.5 m H2SO4	
Pt SAs/MoO_2_	9.3	10	28.78	1.1 wt%	0.5 m H2SO4	[136]
	81.3	100	—		0.5 m H2SO4	
	—	1000	—		0.5 m H2SO4	
	14	10	36.86		1.0 m KOH	

## 4 Interaction between Different Metal‐Based Components of UPLEs

Recently, transition metals (TMs), transition metal oxides (TMOs), transition metal hydroxides (TMOHs), and transition metal phosphides (TMPs), have become indispensable assistants of Pt electrocatalysts for HER applications. The interaction and electronic optimization between them have been regarded as the key, which could enhance the HER activity. However, it is almost a black box for researchers when it comes to electronic regulation. Numerous density functional calculations (DFT) have been carried out to reveal the most possible scenarios.

### 4.1 With TM

**Figure 13:**
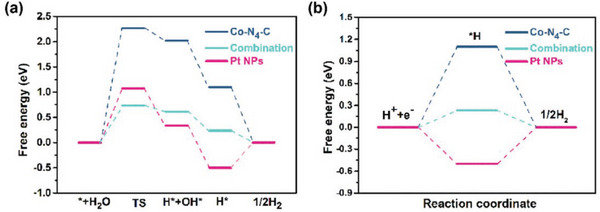
Calculated energy barriers of HER on different active sites of ZIF‐67‐Pt/RGO a) in alkaline, b) in acid. Reproduced with permission.^[^ ^137^ ^]^ Copyright 2020, American Chemical Society.

The combination of Pt with transition metals (TMs) can modulate the d‐band center and promote the absorption and desorption process of H^+^ and OH^−^. So far, transition metal‐based compounds have shown extraordinary catalytic activity for the HER.^[^ ^18^, ^137^, ^138^ ^]^ They could adjust the electronic structure of Pt by interacting, which could also achieve the effect of reducing the Pt loading and improving the catalytic activity. As shown in **Figure** **13**, to further explore the role of these potential active sites (Pt NPs and Co‐N_4_‐C) on the HER catalytic performance, Sun and coworkers calculated the energy barrier of water dissociation on different sites.^[^ ^137^ ^]^ The energy for H—OH bond cleaving was down to 0.73 eV for the combination of Pt NPs and Co‐N_4_‐C, which was much lower than single Pt NPs (1.07 eV) and single Co‐N_4_‐C (2.26 eV). The drop in the water dissociation energy implies that this combination can increase the HER kinetics of the catalyst in an alkaline environment, as the Volmer step becomes the rate‐limiting step (Figure 13a). In addition, this combination showed a more moderate hydrogen binding energy of 0.23 eV, indicating that the interaction between Pt NPs and Co‐N_4_‐C could also improve the HER performance in acidic media (Figure 13b).

**Figure 14:**
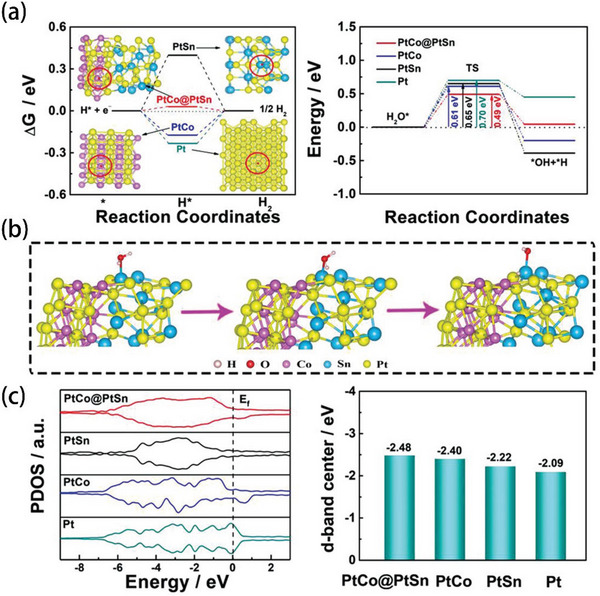
a) Δ*G*_H*_ and TS energy calculated at different adsorption sites for all catalysts, insets: schematic illustration of H adsorption, and reaction energy diagram of water dissociation of all samples. b) Reaction energy diagram of water dissociation of all samples. c) PDOS analysis of Pt and d‐band center values of all samples. Reproduced with permission.^[^ ^18^ ^]^ Copyright 2021, Wiley‐VCH.

Yin et al. used DFT calculations to reveal the underlying mechanism for the improvement of HER activity.^[^ ^18^ ^]^ In **Figure** **14**a,b, PtCo@PtSn delivered the lowest Δ*G*_H*_ (0.03 eV), comparing with PtCo (−0.174 eV), PtSn (0.398 eV), and Pt (−0.233 eV), which indicated that the interaction between PtCo and PtSn alloys can modify the HER catalytic performance. Besides, the barrier for water dissociation of PtCo@PtSn was 0.49 eV, lower than PtCo (0.61 eV), PtSn (0.65 eV), and Pt (0.7 eV), suggesting that alloying with Co and Sn could accelerate water splitting. The projected density of states (PDOS) was additionally carried out to investigate the d‐band center of the catalyst. Figure 14c illustrates that the d‐band of Pt has been adjusted by Co and Sn. As already known, a lower d‐band center indicates more electrons filled in the antibonding state, which could accelerate the desorption of H* from Pt active sites.

### 4.2 With TMOs and TMOHs

**Figure 15:**
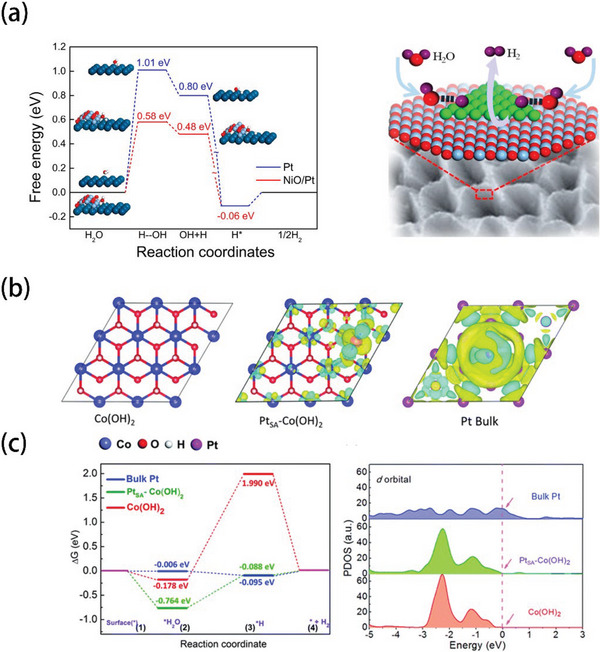
a) Calculated free energy diagram for H_2_O dissociation and H_2_ desorption on the Pt surface (blue) and NiO/Pt interface (red). Insets show the optimized structures at different reaction stages. The large dark blue, small light blue, red, and white balls represent Pt, Ni, O, and H atoms, respectively. Reproduced with permission.^[^ ^139^ ^]^ Copyright 2018, American Chemical Society. b) Top view of the slab models of Co(OH)_2_ and the top view of the calculated electron density differences of the Pt atom in Pt_SA_–Co(OH)_2_, and bulk Pt. c) Calculated adsorption energies of H* and H_2_O on the surface of Co(OH)_2_, Pt_SA_–Co(OH)_2_ and bulk Pt, and the calculated PDOS of d orbitals of bulk Pt, Pt_SA_–Co(OH)_2_ and Co(OH)_2_. Reproduced with permission.^[^ ^64^ ^]^ Copyright 2020, Royal Society of Chemistry.

Transition metal oxides and transition metal hydroxides (TMOs and TMOHs) are often used to accelerate the Volmer step, due to their strong affinity with OH^−^. Meanwhile, the interaction between TMOs/TMOHs and Pt can also optimize the electronic structures of Pt. Wang et al. prepared highly dispersed Pt on NiO@Ni films, then carried out DFT calculations to shed some light on the interaction between Pt and NiO.^[^ ^139^ ^]^ **Figure** **15**a shows that the calculated free energies for H_2_O dissociation and H_2_ desorption of Pt and NiO/Pt. It is obvious that the dissociation energy barrier on NiO/Pt was reduced, highlighting the role of NiO and interaction between Pt and NiO for hydrogen generation. This result suggested that the Volmer step on NiO/Pt surface will no longer be sluggish. Wang's team used DFT calculations to show the electron could be transferred between Co(OH)_2_ and Pt_SA_ (Figure 15b).^[^ ^64^ ^]^ Furthermore, Pt_SA_–Co(OH)_2_ catalyst showed the smallest energies barrier of adsorbing H* (−0.088 eV), and the d‐band was also modified, as shown in Figure 15c. These results implied that the electron cloud gathered around the top of the Pt regions could be useful to accelerate HER kinetics.

### 4.3 With TMPs

**Figure 16:**
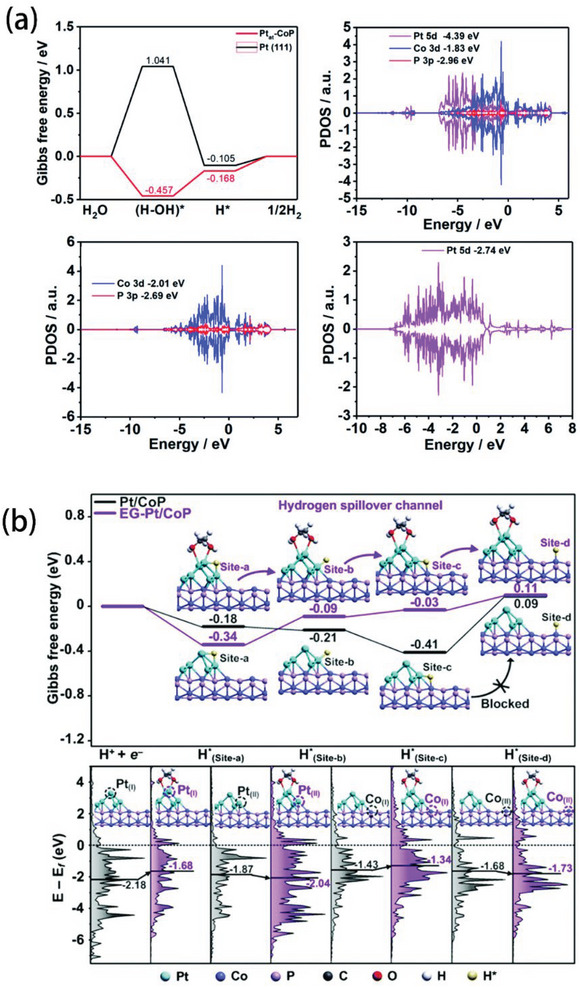
a) Gibbs free energy diagram of the HER process on Pt_at_–CoP and Pt (111) facets; and the PDOS and band centers of Pt 5d, Co 3d, and P 2p in Pt_at_–CoP; Co 3d and P 2p in CoP; Pt 5d in Pt (111) facts. Reproduced with permission.^[^ ^140^ ^]^ Copyright 2020, Royal Society of Chemistry. b) Calculated free energy diagram for hydrogen spillover on Pt/CoP (gray) and EG‐Pt/CoP (purple); Insets are the optimized H* adsorption structures at various sites, and the partial DOS plots of Pt/CoP (gray) and EG‐Pt/CoP (purple); black line shows the position of d‐band center. Reproduced with permission.^[^ ^141^ ^]^ Copyright 2019, Royal Society of Chemistry.

Many studies have shown that phosphorus has excellent electrocatalytic of HER when forming transition metal phosphides (TMPs) with Co, Ni, Mo, and other transition metals.^[^ ^64^, ^139^, ^140^, ^141^, ^142^, ^143^, ^144^, ^145^ ^]^ At the same time, the lattice parameter of phosphide is very close to that of Pt, so it is possible to combine the two to reduce catalyst cost. Computational studies have shown that P atoms exhibit electronegativity and could attract electrons from metal atoms to form electronegative P atoms. Liu and his team calculated the free energy diagram of HER process on the surface of Pt_at_‐CoP and Pt (111) facets.^[^ ^139^ ^]^ In the first step of water splitting, the free energy on Pt_at_‐CoP was −0.457 eV, which tended to be an exothermic process (**Figure** **16**a). On the contrary, the kinetics of water adsorption on Pt (111) facets were notably sluggish, with an energy barrier of 1.041 eV. Also, in Figure 16a, the free energy of H* adsorption was slightly influenced by CoP, 0.168 eV for Pt_at_‐CoP, close to Pt (111) facets (0.105 eV). The interaction between Pt_at_ and CoP was further explained by PDOS: the d‐band center of Pt in Pt_at_‐CoP (−4.39 eV) was downshifted compared with Pt (111) facets. Additionally, 5d orbitals of Pt were hybridized with 2p orbitals of P (Figure 16a). These results suggested that the outstanding HER performance of Pt_at_‐CoP was originated from the interaction of Pt_at_ and CoP, turning H_2_O dissociation to be thermodynamically spontaneous. Qu et al. reported a strategy to further improve the HER performance by using the ethylene glycol (EG).^[^ ^140^ ^]^ In the case of the Pt/CoP catalyst, H* adsorption preferentially happened around the Pt (site a: Pt‐Pt bridge, −0.18 eV; site b: Pt‐top, −0.21 eV, and site c: Pt‐Co bridge, −0.41 eV) instead of CoP (site d, 0.09 eV). Thus, during the HER process of Pt/CoP catalyst, a huge energy barrier between Pt sites to CoP sites hindered the transition of adsorbed H*, indicating the limited efficiency of hydrogen generation. After introducing EG, Δ*G*_H*_ of site a–d changed significantly (Figure 16b). More antibonding electronic states were filled with farther d‐band center from the Fermi Level (0 eV), which weakened the adsorption of H*. The reduced barrier between Pt and CoP sites promoted the transmission of H* and the catalytic performance of HER. Furthermore, moderate interaction of P—H bond optimizes the HER activity and stability of TMPs. However, these electrocatalysts, loading TMPs directly on carbon carriers, still suffer from the checked charge transmission and the limited active surface due to the stacked TMPs on the surface of carbon carriers. In this respect, TMPs can be grown in situ on the metal‐based foams or loaded dispersedly on porous carriers, which can help TMPs distributed uniformly on the carrier and avoid stacking during nucleation. Additionally, incorporation of noble metals (such as Pt) can modify the surface electronic structure of TMPs, modulating the water dissociation energy and H* adsorption.

As for the common DFT calculations for UPLEs can provide insight into the mechanism of the HER reaction. The adsorption energy is the energy required to adsorb a hydrogen atom onto a Pt surface. Lower adsorption energy barrier indicates better HER activity. The density of states and band structure can provide how the charges transfer from other components of UPLEs to Pt and enhance the desorption of hydrogen intermediates. Moreover, the reaction pathways can be used to calculate the energy barriers for the HER on Pt active sites. This can provide insight into the rate‐limiting step of the reaction and identify ways to improve the HER activity.

In conclusion, Pt can interact with TMs, TMOs, TMOHs, and TMPs in different ways depending on the specific conditions of the interaction. Here are some of the essential differences in their interactions. When Pt interacts with other TMs, it can form solid solutions, alloys, and intermetallic compounds. Pt‐TM alloys have high catalytic activity and can be used in various applications such as fuel cells and catalytic converters. The strength of the Pt‐TM interaction depends on the electronegativity and atomic size of the TM, and it can affect the morphology and structure of the resulting materials. When Pt interacts with TMOs, it can form oxide‐supported Pt nanoparticles. These materials have high catalytic activity and selectivity for various reactions, including hydrogenation and oxidation. The interaction between Pt and TMOs depends on the nature of the oxide support, the size and shape of the Pt nanoparticles, and the surface area of the oxide support. When Pt interacts with TMOHs, it can catalyze various reactions such as hydrogenation and dehydrogenation. The interaction between Pt and TMOHs depends on the pH of the solution, the nature of the hydroxide, and the oxidation state of the Pt. When Pt interacts with TMPs, it can form intermetallic compounds with high catalytic activity and selectivity for various reactions such as hydrogenation and dehydrogenation. The interaction between Pt and TMPs depends on the electronegativity and atomic size of the phosphide and the oxidation state of the Pt. Overall, the interactions between Pt and other transition metals, oxides, hydroxides, and phosphides can lead to a wide range of materials with unique properties and applications. The specific nature of the interaction depends on various factors such as the chemical composition, structure, and surface properties of the materials involved.

## 5 Summary and Perspective

**Figure 17:**
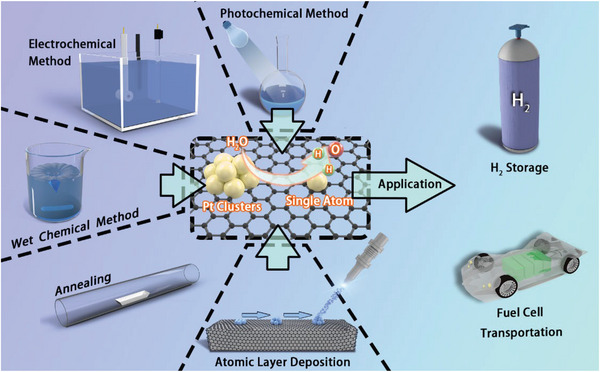
Schematic diagram of different UPLEs synthetic routes.

As shown in **Figure** **17**, hydrogen generation from the water electrolysis is undoubtedly a promising technology for green hydrogen production, and UPLEs could achieve a great balance between the low cost and high HER catalytic activities. In this review, we introduced several synthetic routes of UPLEs reported over the last few years. Here, it is worth noting that the preparation method is not static. The design of catalysts should be highlighted, including components and structures. Carriers also play important roles in designing UPLEs. Those with high specific surface area, rich pore structure, or rich defects can anchor Pt and accelerate HER kinetics (from water dissociation, H* adsorption to hydrogen desorption) through interactions. Meanwhile, a part of the Pt in UPLEs exist in the form of single atoms or clusters, which maximizes the AUE. Overall, the development of UPLEs for HER is a multidisciplinary field that involves a combination of experimental synthesis and characterization, theoretical modeling and simulation, and engineering design and optimization. Ongoing research in this area holds great promise for advancing the state‐of‐the‐art in electrocatalysis and enabling the widespread deployment of sustainable hydrogen production technologies.

Although current findings are promising, more efforts should be focused on the following areas:

1. Since the study of structural changes at an atomic scale is crucial for understanding the mechanism of the interaction between active sites during the HER process, the most effective method so far is the Extended X‐ray absorption fine structure (EXAFS) measurement, which is not widely available. Further, the design of an in situ cell for X‐ray absorption fine structure (XAFS) measurements during electrochemical testing is a specialized area of research which is still need to be modified. The main goal of an in situ cell design for XAFS measurements during electrochemical testing is to allow the sample to be exposed to a controlled electrochemical environment while maintaining good signal‐to‐noise ratio in the XAFS spectra. The in situ cell should have transparent windows made of materials, allowing X‐rays to pass through the sample. Also, it must be hermetically sealed to prevent contamination of the electrolyte or gas by air or moisture. O‐rings or epoxy seals are commonly used for sealing. If the sample is exposed to gas during the electrochemical testing, the in situ cell must be gas‐tight to prevent leakage of the gas. Overall, the design of an in situ cell for XAFS measurements during electrochemical testing is a complex process that requires careful consideration of many factors, including the nature of the sample, the electrochemical conditions, and the X‐ray absorption edge of interest.
2. Many researchers have provided feasible ideas and information for HER through DFT, XRD, TEM, and XPS, etc. Among those characterization techniques PDF (Pair Distribution Function) analysis is a mathematical technique that uses X‐ray or neutron scattering data to determine the distribution of atoms in a material on a local scale. By analyzing the way that X‐rays or neutrons scatter off of the atoms in a material, PDF analysis can provide information about the distances between atoms and the way that they are arranged in the material. PDF could be a useful tool to investigate the structure of electrocatalysts before and after electrochemical testing. Electrocatalysts are materials that are used to catalyze chemical reactions that take place during electrochemical processes, such as fuel cells, batteries, and electrolysis cells. During these processes, the electrocatalysts can undergo changes in their structure and composition, which can affect their catalytic activity and durability. PDF analysis can provide valuable information about the local atomic structure of electrocatalysts, such as the distribution of metal atoms, the coordination of metal ions with ligands, and the presence of defects or disorder. By analyzing the changes in the PDF patterns of electrocatalysts before and after electrochemical testing, researchers can gain insights into the structural changes that occur during the electrochemical process. Moreover, other interfacial structure characterization techniques to observe the real‐time changes of active sites during electrochemical processes are urgently required. For example, the in situ infrared spectroscopy could be used to characterize the adsorption status of groups on the catalyst surface or using in situ Raman spectroscopy to observe the changes in surface metal sites during electrocatalysis.
3. The stability of Pt‐based metal electrocatalysts is still one of the research hotspots in recent years. In the process of hydrogen generation, especially under acidic conditions, the Pt loading on the working electrode could more easily be dissolved and reduced, which greatly affects the stability of Pt‐based catalysts. Therefore, forming ordered alloys with transition metals is a feasible strategy to improve the stability of Pt‐based catalysts. In alkaline solution, Pt will slightly be dissolved and reduced during the hydrogen evolution, but the huge energy barrier of water dissociation remains to be a problem. Here, combining with more stable transition metal derivatives should be useful. However, many work need to be done to develop the Pt‐based catalysts for a wide‐range pH value.
4. To realize the commercial application of hydrogen energy, a strategy suitable for large‐scale production should be provided, being originated from the existing strategies summarized above. For industrial applications, the durability usually counts for months or even years, far exceeding the current reported testing time. Moreover, UPLEs should be improved to be robust and highly efficient under a high current density. To this end, how to protect the active sites from migration or aggregation during long‐term water electrolysis means a lot to the commercialization process. In addition to H_2_ generation, Pt‐based catalysts have also been widely used in ORR and methanol/ethanol oxidation reactions. Usually, 1–5 nm Pt clusters or Pt single atoms can be obtained in UPLEs. However, the precise control of Pt‐based electrocatalysts is only in the laboratory‐scale. How to mass‐produce UPLEs for commercial fuel cells such as direct methanol fuel cells and proton exchange membrane fuel cells is still a technical bottleneck.

## Conflict of Interest

The authors declare no conflict of interest.
